# Effectiveness of Herbal Interventions in the Management of Hypercholesterolemia: Protocol for a Systematic Review

**DOI:** 10.2196/68016

**Published:** 2025-11-19

**Authors:** Anju Aravind, Athul TP, Ribin V P, Deenadayal Devarajan, Anagha M S, Robin Badal, Akanksha Thakur, Krishna Raghava Gangotri, Meera K Bhojani

**Affiliations:** 1 All India Institute of Ayurveda New Delhi India; 2 Prasanna College of Ayurveda & Hospital Mangalore India

**Keywords:** herbal interventions, hypercholesterolemia, systematic review, elevated cholesterol levels, herbal medicines

## Abstract

**Background:**

Hypercholesterolemia is a significant risk factor for cardiovascular diseases, necessitating effective management strategies. Herbal interventions have gained attention as potential alternative or complementary therapies to conventional lipid-lowering medications.

**Objective:**

This systematic review aims to evaluate the effectiveness of various herbal interventions in managing hypercholesterolemia.

**Methods:**

A comprehensive literature search will be conducted across multiple databases, including PubMed, MEDLINE, EMBASE, EBSCO, Cochrane Library, DHARA, AYUSH research portal, WHO portal, Shodhganga, and Google scholar as well as dissertations and thesis work available in the public domain and unpublished works from university websites for studies published from January 2020 onward. Randomized controlled trials investigating the impact of herbal interventions on cholesterol levels will be included. The primary outcome is the change in low-density lipoprotein cholesterol and high-density lipoprotein cholesterol levels. Data extraction and quality assessment will be performed independently by 3 reviewers, and discrepancies will be resolved by a fourth reviewer. All statistical analyses will be conducted using the Cochrane Collaboration software program, RevMan Web.

**Results:**

Following an initial screening, 96 studies were identified. After removing duplicates, 7 studies were excluded, leaving 89 studies ready for data extraction. The results of these studies will be synthesized and analyzed upon completion.

**Conclusions:**

This review aims to synthesize evidence on the potential benefits of herbal interventions in managing hypercholesterolemia. Preliminary findings suggest that specific herbal interventions may contribute to lower cholesterol levels, potentially complementing standard hypercholesterolemia management strategies. The findings will be systematically analyzed and presented upon completion of the review, providing insights into the effectiveness of integrating these interventions into current treatment protocols.

**Trial Registration:**

PROSPERO CRD42024548858; https://www.crd.york.ac.uk/PROSPERO/view/CRD42024548858

**International Registered Report Identifier (IRRID):**

DERR1-10.2196/68016

## Introduction

Lipoproteins are complexes of lipids and proteins essential for transporting cholesterol, triglycerides, and fat-soluble vitamins in the blood. Plasma lipoproteins are classified into 5 major classes based on their relative density: chylomicrons, very low-density lipoproteins, intermediate density lipoproteins, low-density lipoproteins (LDL), and high-density lipoproteins (HDL). Key roles of lipoproteins are to transport dietary lipids from the intestines to tissues requiring fatty acids for energy storage and metabolism and to transport intestinal cholesterol to the liver.

Disorders of lipoprotein metabolism, known as dyslipidemias, are clinically characterized by increased plasma levels of cholesterol, triglycerides, or both [1]. The etiological classification of dyslipidemia is categorized as primary (genetic) or secondary (lifestyle and other). Hypercholesterolemia falls under the primary type of dyslipidemia.  Hypercholesterolemia is a polygenic disorder characterized by an increase in cholesterol, whereas in hypertriglyceridemia there is an increase in triglycerides [2]. Plasma lipoprotein levels are significant modifiable risk factors for cardiovascular diseases, with increased levels of LDL contributing to the development of atherosclerosis [3].

According to the Global Health Observatory, raised cholesterol is estimated to cause 2.6 million deaths globally and 29.7 million (2%) disability-adjusted life years [4]. Elevated total cholesterol is a significant cause of disease burden in both high- and low-income countries, serving as a risk factor for ischemic heart disease and stroke [4]. Very low-density lipoproteins and triglycerides contribute to the building up of arterial plaque.

Factors such as genetics, high intake of saturated fats, sedentary lifestyle, and certain medical conditions, including diabetes and hypothyroidism, can lead to hypercholesterolemia. Modification of lifestyle, including changes in food habits, cessation of smoking, reduction of alcohol intake, and regular exercise, is not only necessary to attain normal lipid levels but also represents the very first step in the management of hypercholesterolemia [3]. Standard treatments include statins, ezetimibe, bile acid sequestering resins, proprotein convertase subtilisin/kexin type 9 inhibitors, and nicotinic acid [4].

Studies have shown that garlic [5-7], *Triphala* [8], Nigella sativa seeds [9], and Terminalia arjuna [10] have significant lipid-lowering effects and are effective in managing dyslipidemia. Garlic reduces serum lipid levels, while *Triphala* and Nigella sativa improve lipid profiles safely. Terminalia arjuna also lowers total and LDL cholesterol with antioxidant benefits comparable to vitamin E, making these natural agents promising for cholesterol management. Additionally, there is growing interest in herbal medications as alternative or complementary treatments, as these natural remedies may offer potential benefits in managing cholesterol levels. This systematic review will aim to assess the effectiveness of herbal interventions in hypercholesterolemia.

## Methods

### Overview

This review protocol was prepared and reported in accordance with the PRISMA-P (Preferred Reporting Items for Systematic Review and Meta-Analysis Protocols) guidelines and the Cochrane Handbook for Systematic Reviews of Interventions. The trial has been registered in PROSPERO (CRD 42024548858).

### Types of Interventions

#### Overview

The review will include studies on single or polyherbal drugs in any form and dosage, including those combined with dietary regimens, physical activity, or both. Studies evaluating single or polyherbal drugs alongside the standard of care for hypercholesterolemia will be included. If herbal interventions are combined with other treatments such as diet or physical activity, these studies will be categorized as combination therapy and analyzed separately to assess the specific contribution of herbal interventions. In cases where herbal interventions are part of a combination therapy, a hierarchical approach will be used. Priority will be given to the analysis of outcomes directly attributed to the herbal component, if reported separately. Otherwise, the overall effect of the combined intervention will be considered.

#### Inclusion Criteria

The following studies will be included: (1) randomized controlled trials; (2) systematic reviews and meta-analyses providing relevant data (these will be considered for background information); (3) studies involving adult participants (aged ≥18 years, regardless of sex) diagnosed with hypercholesterolemia or dyslipidemia as per established clinical criteria; (4) studies involving populations with secondary causes of dyslipidemia (eg, diabetes or hypothyroidism) if data specific to hypercholesterolemia are provided; (5) studies evaluating the effects of single or polyherbal formulations, whether in raw, processed, or extract forms; and (6) full-text articles published in any language.

#### Exclusion Criteria

The following studies will be excluded: (1) studies with insufficient methodological details or unclear reporting of outcomes; (2) nonrandomized controlled trials, animal trials, case series, commentaries, letters to the editor, case reports, narrative reviews, editorials, abstracts, meetings, and studies with insufficient information on herbal interventions and hypercholesterolemia; and (3) research papers with study durations less than 4 weeks.

#### Comparison or Control Groups

Control groups involving placebo, no intervention, diet, exercise, standard of care, or any combination of these will be considered. If a trial includes multiple arms, any arm that meets the inclusion criteria will be included in the review.

#### Outcome Measures

Primary outcomes include LDL cholesterol levels (>130 mg/dl) and HDL cholesterol levels (<40 mg/dl). Additional outcomes include cardiovascular events (high blood pressure, stroke, or coronary artery disease), triglycerides (>150 mg/dl), total cholesterol (>200 mg/dl), and changes in BMI and body weight.

### Search Methods for Study Identification

The literature search will be conducted across multiple databases, including the Cochrane Central Register of Controlled Trials (CENTRAL), MEDLINE, EMBASE, EBSCO, AYUSH research portal, DHARA, Google Scholar, and Shodhganga. We will search clinical trial registries for ongoing and recently completed trials (eg, ClinicalTrials.gov, World Health Organization International Trials Registry and Platform, and ISRCTN Registry). The search strategy will combine Medical Subject Headings terms and free text words using the Boolean operators “AND” and “OR.” For example, “hypercholesterolemia,” “increased cholesterol levels,” “dyslipidemias,” “hyperlipidemias,” “increased lipid levels” AND “herbal interventions,” “herbal medicines,” “plant extracts,” “medicinal plants,” “herbs,”  “Chinese herbal drugs,” “Mongolian traditional medicine,” “Ayurveda,” “Ayurved,” “East Asian traditional medicine,” “medicine,” “biological products”. Different search strategies will be used for different databases.

Dissertation and thesis work available in the public domain as well as unpublished postgraduate and doctoral dissertations from university and institutional websites and other potential sources from inception to present will be included. We will use backward and forward citation searching to identify additional relevant papers. Searches will be rerun prior to initiating the final analysis. If published data for a registered trial cannot be found, we will contact the principal investigator via email to obtain the trial data.

### Data Collection and Analysis

#### Selection of Studies

Four review authors (AAT, RVP, AMS and ATP) will independently screen the titles and abstracts of articles identified through the searches to determine suitability. Studies will be classified as included, unclear, or excluded. For studies without an abstract or with a limited abstract, full articles will be retrieved and assessed for inclusion. Three review authors will independently evaluate the full articles using a standardized form with explicit inclusion and exclusion criteria. Any discrepancies will be resolved through consultation with a fifth review author (MKB). The selection of studies will follow the PRISMA guidelines [11].

#### Data Extraction

The data extraction form will include the following in accordance with the standards prescribed in the PRISMA guidelines: author and year of publication, study setting, study population, participant demographics, study methodology, details of the intervention and comparator, outcome measures, and study results.

#### Missing Data

If there is incomplete information or a lack of data, we will contact the author by telephone or email. If the missing data cannot be obtained, we will use replacement values to fill in the gaps, if necessary, or consult with experts.

#### Data Synthesis and Assessing Heterogeneity

All statistical analyses will be conducted using RevMan Web version 5.3 software (Cochrane Collaboration). A *P* value of <.05 will be considered statistically significant between studies. Heterogeneity will be assessed using the *I*² statistic. RevMan Web is widely recognized as a comprehensible tool specifically designed for conducting systematic reviews and meta-analyses. Its ability to handle heterogeneous data through random-effects models, subgroup analysis, and forest plots makes it particularly suitable for this study.

#### Subgroup Analysis

If the clinical trials cause heterogeneity, we will perform a subgroup analysis based on interventions, controls, durations of interventions, and outcome measurements. Adverse reactions will be tabulated and then evaluated. Subgroup analyses will be performed to evaluate the effectiveness of single herbal interventions in comparison to polyherbal interventions as well as the impact of herbal therapies used alone compared to those combined with other lifestyle modifications (eg, diet or exercise).

#### Risk of Bias in Individual Studies

Four review authors (RB, DD, KRG, and AK) will independently assess the risk of bias of each included trial. We will resolve any disagreements by consensus or by consultation with a fifth review author (MKB). If adequate information is not available from trial authors, trial protocols, or both, we will contact trial authors for missing data on risk of bias items. Randomized controlled trials will be assessed by using the Cochrane Risk of Bias tool–2, which classifies studies as having some concern, low risk of bias, or high risk of bias by evaluating domains like randomization process, deviation from the intended interventions, missing outcome data, measurement of the outcome, and selection of the reported results [12]. The risk of bias findings will be presented as a summary and graph.

#### Assessment of Publication Bias

Potential publication bias will be addressed by including studies published in all languages and considering both published and unpublished data.

#### Assessment of Strength of Evidence

The strength of evidence will be assessed using the GRADE (Grading of Recommendations, Assessment, Development, and Evaluations) approach. GRADE evaluates the quality of evidence based on certain factors. Risk of bias will be assessed for methodological quality and different types of biases of studies. Inconsistency will be assessed by considering the variability of results across studies. Indirectness will be evaluated by analyzing the applicability of the evidence to the research question. Imprecision will be estimated by assessing the precision of effect estimates based on confidence intervals and sample sizes.

### Data analysis

#### Descriptive Analysis

The review will summarize the characteristics of the included studies, such as author, year of publication, study design, sample size, population characteristics, and duration of the intervention. Tables and narrative summaries will be used to present the types of herbal interventions used, including dosages and comparison groups. Additionally, the baseline characteristics of participants, such as age, gender, baseline lipid levels, and comorbidities, will be summarized in tables or using descriptive statistics like means, medians, ranges, and standard deviations.

#### Statistical Analysis

To analyze primary outcomes such as HDL and LDL levels, we will calculate the mean difference or standardized mean difference for these continuous variables between intervention and control groups. We will choose between random-effects or fixed-effects models based on the level of heterogeneity, which can be assessed using the *I*² statistic. For dichotomous outcomes such as the incidence of cardiovascular events, we will use relative risk or odds ratios for the analysis. Similar to HDL and LDL levels, we will calculate the mean difference or standardized mean difference for continuous outcomes like triglyceride and total cholesterol levels. To analyze changes in BMI and body weight, we will calculate the mean difference or standardized mean difference. Additionally, we will consider performing subgroup analyses to explore differences based on the type of herbal intervention.

### Ethical Considerations

Ethical approval was not required as systematic reviews are a form of secondary research and the review did not include any confidential information about participants.

## Results

Following an initial screening process, a total of 96 studies were identified as relevant. A duplicate-checking procedure was then conducted, resulting in the removal of 7 duplicate studies, with 89 unique studies remaining eligible for further review and data extraction. These studies will undergo a detailed synthesis and analysis to assess key findings, trends, and implications. Data collection began on November 1, 2024.

## Discussion

Hypercholesterolemia, a condition characterized by elevated levels of cholesterol in the blood, is a significant risk factor for cardiovascular diseases such as ischemic heart disease and stroke. Recently, there has been growing interest in herbal interventions as alternative or complementary treatments for managing cholesterol levels. Herbal interventions typically exert their cholesterol-lowering effects through various mechanisms. A meta-analysis on garlic concluded that garlic, in powder or nonpowder form, can significantly lower serum lipid levels over a 1- to 3-month period [5], and a clinical trial conducted to evaluate efficacy concluded that the essential oil of garlic exerts a distinct hypolipidemic action in both healthy individuals and patients of coronary heart disease [6]. Additionally, a study examining the effects of garlic on obesity and blood lipid profiles in high-fat diet–induced obese mouse models revealed that administration of garlic significantly reduced high-fat diet–induced weight gain, epididymal fat accumulation, and hypercholesterolemia [7]. However, evidence from a meta-analysis on the effect of garlic on serum cholesterol suggests that the intake of garlic preparations did not produce any significant beneficial effects on serum cholesterol levels [13]. An animal study on the *Curcuma longa* revealed that while lipid accumulation significantly increased in the free fatty acids–treated group compared to the control group, supplementation with the water extract of *Curcuma longa* effectively reduced lipid accumulation in a dose-dependent manner, demonstrating its potential for regulating lipid metabolism [14]. A meta-analysis on *Curcuma longa* suggested that curcumin (ie, turmeric) supplementation may be effective in improving blood levels of total cholesterol, triglycerides, LDL cholesterol, and HDL cholesterol, but may not be capable of improving their pertinent apolipoproteins [15]. A randomized clinical study was conducted to evaluate the efficacy and safety of *Triphala*, an Ayurvedic powder formulation consisting of Terminalia chebula, Terminalia bellerica, and Emblica officinalis. The study found that *Triphala* is both effective and safe for the management of dyslipidemia [8]. A randomized clinical study was also conducted to evaluate the efficacy of Nigella sativa seeds and concluded that they have beneficial therapeutic effects in the treatment of hyperlipidemia [9]. A randomized controlled trial evaluated the antioxidant and hypocholesterolemic effects of *Terminalia arjuna* tree bark powder in patients with coronary heart disease and found that *Terminalia arjuna* significantly reduced total and LDL cholesterol levels, with its antioxidant effects comparable to those of vitamin E, demonstrating potential therapeutic benefits in managing cholesterol levels [10].

Collating all the above results into a common pool of evidence through a systematic review and meta-analysis will hopefully benefit clinicians focusing on hypercholesterolemia. Comparative findings on alternative therapies targeting this spectrum of disease will undoubtedly contribute to building a robust treatment protocol, ultimately promoting an integrative approach. This systematic review aims to provide comprehensive evidence on the effectiveness and safety of herbal interventions in managing hypercholesterolemia. The findings could guide health care professionals in recommending herbal supplements as part of a holistic treatment approach and inform future research directions.

The main limitation of the study is that most clinical trials conducted in alternative medicine, such as Ayurveda, are not yet available in leading journals indexed in major databases. Additionally, clinical trials with positive results, conducted as part of academic work, have not been published or made accessible to the public. This limits the availability of high-quality evidence, potentially affecting the comprehensiveness of the review.

The systematic review will be published in a peer-reviewed journal and disseminated both electronically and in print. It will be periodically updated, and a GRADE 2.0 evaluation will be conducted to assess the quality of evidence, providing summaries on the future state of the research.

## Abbreviations

CENTRAL: Cochrane Central Register of Controlled Trials
GRADE: Grading of Recommendations, Assessment, Development, and Evaluations
HDL: high-density lipoprotein
LDL: low-density lipoprotein
PRISMA-P: Preferred Reporting Items for Systematic Review and Meta-Analysis Protocols

## Data Availability

This is a study protocol. The data will be publicly available after the systematic review is published.
